# Supplementary material for: Disparities in COVID-19 incidence and fatality rates at high-altitude
Source: PeerJ. 2023 Feb 6;11:e14473. doi: 10.7717/peerj.14473 (PMC9922493; doi:10.7717/peerj.14473)
Supplement: Supplemental Information 1 [file peerj-11-14473-s001.docx]

**Supplementary Table 1:** COVID-19 literature with altitude-associated different geographies.

| **Ref. No.** | **Literature** | **Threshold used**  **(LA.)** | **Threshold used**  **(HA.)** | **Findings** |
| --- | --- | --- | --- | --- |
|  | **Protection** | | | |
| 01 | Impact of altitude on COVID-19 infection and death in the United States: A modeling and observational study. | LA <914masl | HA>2133masl | Lower cases at HA vs. LA i.e. 615, vs 905, respectively, p=0.034) |
| 02 | Does the pathogenesis of SARS-CoV-2 virus decrease at high-altitude? | Not provided | >2500 masl | Denoted a lower incidence rate at high-altitude. |
| 03 | Negative Correlation between Altitude and COVID-19 Pandemic in Colombia: a preliminary report. | Not provided | Not provided | Negative relation was observed between Altitude and COVID-19. |
| 04 | Altitude conditions seem to determine the evolution of COVID-19 in Brazil | LA ≤ 97 masl  Moderate Altitude(97 ≤x<795) masl | HA (795 ≤x ≤1135 masl) | HA cities may be favorable to shelter people at risk. |
| 05 | Propagation by COVID-19 at high altitude: Cusco case | Not provided | Not provided | HA may provide protection, but the additional evaluation is needed. |
| 06 | Factors involved in low susceptibility to COVID-19: An adaptation of high altitude inhabitants | Not provided | >2500 masl | ACE-2 expressions, Activation of HIF, and greater lung capacity may assist in the less susceptibility of SARS-CoV-2 in HA Inhabitants. |
| 07 | Hypoxia inducible factor-1 protects against COVID-19: A hypothesis | Not provided | Not provided | HIF-1 stabilization could assist in lowering COVID-19 infection rates |
| 08 | High levels of ambient ozone (O3) may impact COVID-19 in high altitude mountain environments | Not provided | Not provided | Ozone, along with other ecological factors, could assist in the understanding of the infection rate at HA. |
|  | **Risk** | | | |
| 09 | Morality attributed to COVID-19 in High Altitude Populations. | Ranges:  0-1500masl  1500-1999masl  >2000masl | >1500 masl | Altitude is associated with COVID-19 mortality in men younger than 65 years |
| 10 | Letter to the editor: Influence of Altitude in the Prevalence and Case Fatality rate of COVID-19 in Peru | Not provided | >2500 masl | Not provided |
| 11 | Lower Incidence of COVID-19 at High Altitude: Facts & Confounders | Not provided | >2500 masl | There is currently little supporting evidence for any protective benefit of genetic or non-genomic adaptation to HA hypoxia, including the concept that hypoxia-mediated alterations in ACE-2 expression or ACE-2 variants in particular populations. Cohorts might have relevance to the pathogenesis or severity of the disease. |
| 12 | High altitude reduces infection rate of COVID-19 but not case-fatality rate | Not provided | Not provided | COVID-19 infection at HA is lower; Case fatality is not affected. |
| 13 | Altitude and COVID-19: Friend or foe? A narrative review. | Not provided | Not provided | Currently, it appears doubtful that living at altitude has any protective effects against SARS-CoV-2 infection. |
| 14 | Altitude does not protect against SARS‐CoV‐2 infections and mortality due to COVID‐19. | Ranges:  <1500masl, 1500 to <2500 masl | 2500-4700masl. | Altitude, especially over 1500 m, has little protective effect on the virulence or lethality of the SARS-CoV-2 virus. |

masl, metre above sea level; LA, Low-altitude; HA, High-altitude; ACE2, Angiotensin-Converting Enzyme 2

**References Supplementary Table 1:**

1. Stephens K, Chernyavskiy P, Bruns D. Impact of altitude on COVID-19 infection and death in the United States: A modeling and observational study. *PLoS One*. 2021;16(1):e0245055. doi:10.1371/journal.pone.0245055.
2. Arias-Reyes C, Zubieta-DeUrioste N, Poma-Machicao L, et al. Does the pathogenesis of SARS-CoV-2 virus decrease at high-altitude?. *Respir Physiol Neurobiol*. 2020;277:103443. doi:10.1016/j.resp.2020.103443.
3. Cano-Pérez E, Torres-Pacheco J, Fragozo-Ramos M, et al. Negative Correlation between Altitude and COVID-19 Pandemic in Colombia: A Preliminary Report. *Am J Trop Med Hyg*. 2020;103(6):2347-2349. doi:10.4269/ajtmh.20-1027.
4. Fernandes J, da Silva R, Silva A, et al. Altitude conditions seem to determine the evolution of COVID-19 in Brazil. *Sci Rep*. 2021;11(1). doi:10.1038/s41598-021-83971-x.
5. Huamaní C, Velásquez L, Montes S, et al. Propagation by COVID-19 at high altitude: Cusco case. *Respir Physiol Neurobiol*. 2020;279:103448. doi:10.1016/j.resp.2020.103448.
6. Choquenaira-Quispe C, Saldaña-Bobadilla V, Ramirez J. Factors involved in low susceptibility to COVID-19: An adaptation of high altitude inhabitants. *Med Hypotheses*. 2020;143:110068. doi:10.1016/j.mehy.2020.110068.
7. Afsar B, Kanbay M, Afsar R. Hypoxia inducible factor-1 protects against COVID-19: A hypothesis. *Med Hypotheses*. 2020;143:109857. doi:10.1016/j.mehy.2020.109857.
8. Semple J, Moore G. High levels of ambient ozone (O3) may impact COVID-19 in high altitude mountain environments. *Respir Physiol Neurobiol*. 2020;280:103487. doi:10.1016/j.resp.2020.103487.
9. Woolcott O, Bergman R. Mortality Attributed to COVID-19 in High-Altitude Populations. *High Alt Med Biol*. 2020;21(4):409-416. doi:10.1089/ham.2020.0098.
10. Intimayta-Escalante C, Rojas-Bolivar D, Hancco I. Letter to the Editor: Influence of Altitude on the Prevalence and Case Fatality Rate of COVID-19 in Peru. *High Alt Med Biol*. 2020;21(4):426-427. doi:10.1089/ham.2020.0133.
11. Pun M, Turner R, Strapazzon G, et al. Lower Incidence of COVID-19 at High Altitude: Facts and Confounders. *High Alt Med Biol*. 2020;21(3):217-222. doi:10.1089/ham.2020.0114.
12. Segovia-Juarez J, Castagnetto J, Gonzales G. High altitude reduces infection rate of COVID-19 but not case-fatality rate. *Respir Physiol Neurobiol*. 2020;281:103494. doi:10.1016/j.resp.2020.103494.
13. Millet GP, Debevec T, Brocherie F, Burtscher M, Burtscher J. 2021. Altitude and COVID-19: Friend or foe? A narrative review. Physiological Reports 8:e14615. DOI: 10.14814/phy2.14615.
14. Cardenas L, Valverde‐Bruffau V, Gonzales GF. 2021. Altitude does not protect against SARS‐CoV‐2 infections and mortality due to COVID‐19. *Physiological Reports* 9:e14922. DOI: 10.14814/phy2.14922.

**SUPPLEMENTARY TABLE 2:** Sources of the collection of COVID-19 data.

| 1. **COVID-19 and population-level data sources** | | | |
| --- | --- | --- | --- |
|  | **S. No** | **Data type** | **Links/ References** |
|  | 01 | Indian COVID-19 data | https://www.mygov.in/corona-data/covid19-statewise-status |
|  | 02 | International COVID-19 data | <https://coronavirus.jhu.edu/>, <https://www.worldometers.info/coronavirus/> |
|  | 03 | Population level-data | <https://www.citypopulation.de/> <https://www.statista.com/>. |
| 1. **Altitude data sources** | | | |
| 01 Altitude-level data <https://en-in.topographic-map.com/> | | | |
|  |  |  |  |
| 1. **Cofactors and comorbidities sources** | | | |
|  | 01 | BMI, Blood Pressure, Blood sugar, Tobacco, Alcohol consumption, PM2.5 for Indian population | <https://data.gov.in/> NHFS 4-5(2015-2016), Ministry of Health and Family Welfare, Government of India. |
|  | 02 | Hypertension and Diabetes for Indian population | Supplementary Ref**^1-2^** |
|  | 03 | Cardiovascular disease, Asthma, Cancer and Stroke for Indian population | <https://data.gov.in/> NHFS 4-5(2015-2016), Ministry of Health and Family Welfare, Government of India. |
|  | 04 | Global BMI data | Supplementary Ref**^3^** |
|  | 05 | Global Blood Pressure data | <https://ncdrisc.org/data-downloads-blood-pressure.html> |
|  | 06 | Global haemoglobin data | Supplementary Ref**^4-5^** |
|  | 07 | Global Hypertension | Supplementary Ref**^6^** |
|  | 08 | Global Diabetes | <https://ourworldindata.org/> |
|  | 09 | Global alcohol consumption, Age>65, GDP per capita | <https://ourworldindata.org/> |
|  | 10 | Global Cardiovascular death rate | <https://ourworldindata.org/> |

BMI, body mass index, PM2.5, particulate matter < 2.5 micrometres; GDP, gross domestic product; Indian population level data were based on census 2011

**SUPPLEMENTARY TABLE 2 References:**

1. Geldsetzer P, Manne-Goehler J, Theilmann M, et al. Diabetes and Hypertension in India. *JAMA Intern Med*. 2018;178(3):363. doi:10.1001/jamainternmed.2017.8094.
2. Tandon N, Anjana R, Mohan V, et al. The increasing burden of diabetes and variations among the states of India: the Global Burden of Disease Study 1990–2016. *The Lancet Global Health*. 2018;6(12):e1352-e1362. doi:10.1016/s2214-109x(18)30387-5.
3. Kelly T, Yang W, Chen C, et al. Global burden of obesity in 2005 and projections to 2030. *Int J Obes*. 2008;32(9):1431-1437. doi:10.1038/ijo.2008.102.
4. Gassmann M, Mairbäurl H, Livshits L, et al. The increase in hemoglobin concentration with altitude varies among human populations. *Ann N Y Acad Sci*. 2019. doi:10.1111/nyas.14136.
5. Bazo-Alvarez J, Quispe R, Pillay T, et al. Glycated haemoglobin (HbA1c) and fasting plasma glucose relationships in sea-level and high-altitude settings. *Diabetic Medicine*. 2017;34(6):804-812. doi:10.1111/dme.13335.
6. Mills K, Bundy J, Kelly T, et al. Global Disparities of Hypertension Prevalence and Control. *Circulation*. 2016;134(6):441-450. doi:10.1161/circulationaha.115.018912.

**SUPPLEMENTARY TABLE 3:** A detailed comparison of HA vs LA populations for the COVID-19 infection incidence, recovery, mortality and fatality.

| **S No.** | **Regions**  **(Countries**  **/County’s**  **/states)** | **Altitude**  **(masl)** | **Population**  **(In millions)** | **Confirmed COVID-19 cases** | **Incidence Rate = Confirmed cases/Total Population*100**  **(%)** | **COVID19 recovered cases** | **Confirmed COVID19 death cases** | **Fatality rate = COVID19 Confirmed Death cases/COVID 19 Confirmed Cases*100**  **(%)** |
| --- | --- | --- | --- | --- | --- | --- | --- | --- |
| **I.** | **INDIA** |  |  |  |  |  |  |  |
|  | LA1 (n=20) | 0-500 | 769.45 | 8744181 | 1.39±0.23 | 8454171 | 121671 | 1.12±0.12 |
|  | LA2 (n=06) | 501-1000 | 162.95 | 595448 | 0.57±0.12 | 587778 | 5227 | 0.83±0.15 |
|  | LA3 (n=03) | 1001-1500 | 367.63 | 1370838 | 0.48±0.11 | 1333070 | 24959 | 2.12±0.51 |
|  | LA4 (n=01) | 1501-2000 | 35.60 | 217682 | 0.61±0.00 | 214964 | 1094 | 0.50±0.00 |
|  | LA5 (n=02) | 2001-2500 | 12.82 | 114319 | 0.96±0.10 | 111903 | 1751 | 1.03±0.70 |
|  | Total LA (n=32) | < 2500 | 1348.47 | 11042468 | 1.10±0.16 | 10701886 | 154702 | 1.14±0.11 |
|  | HA (n=4) | ≥ 2500 | 22.037 | 202318 | 1.50 ±0.6 | 197508 | 3228 | 1.68±0.18 |
| **II.** | **AFRICAN COUNTRIES** | | | | | | | |
|  | LA1 (n=36) | 0-500 | 759.00 | 3303230 | 0.79±0.22 | 3018759 | 93544 | 2.84±0.42 |
|  | LA2 (n=15) | 501-1000 | 508.69 | 586440 | 0.30±0.13 | 486632 | 12517 | 3.44±0.76 |
|  | LA3 (n=04) | 1001-1500 | 80.01 | 163293 | 0.50±0.33 | 128220 | 1951 | 1.37±0.37 |
|  | LA4 (n=02) | 1501-2000 | 15.33 | 30942 | 0.32±0.16 | 22726 | 591 | 4.68±3.18 |
|  | LA5 (n=0) | 2001-2500 | NA | NA | NA | NA | NA | NA |
|  | Total LA (n=57) | < 2500 | 1363.04 | 4083905 | 0.63±0.15 | 3656337 | 108603 | 2.96±0.35 |
|  | HA (n=0) | ≥ 2500 | NA | NA | NA | NA | NA | NA |
| **III.** | **ARGENTINA** | | | | | | | |
|  | LA1 (n=14) | 0-500 | 36.31 | 2779580 | 7.33±1.38 | 2053543 | 58006 | 1.68±0.09 |
|  | LA2 (n=04) | 501-1000 | 3.95 | 272039 | 8.05±1.28 | 251808 | 5116 | 1.81±0.22 |
|  | LA3 (n=01) | 1001-1500 | 1.71 | 103828 | 6.05±0.00 | 94142 | 1773 | 1.70±0.00 |
|  | LA4 (n=03) | 1501-2000 | 2.63 | 77749 | 3.21±0.41 | 2363 | 68205 | 3.12±0.61 |
|  | LA5 (n=01) | 2001-2500 | 0.41 | 16534 | 3.94±0.00 | 12667 | 117 | 0.70±0.00 |
|  | Total LA (n=31) | < 2500 | 45.02 | 3249730 | 6.71±0.92 | 2479965 | 67375 | 1.85±0.14 |
|  | HA (n=01) | ≥ 2500 | 0.77 | 24973 | 3.2±0.00 | 22033 | 1055 | 4.42±0.00 |
| **IV.** | **CHILE** |  |  |  |  |  |  |  |
|  | LA1 (n=05) | 0-500 | 2.45 | 163205 | 8.78±1.93 | 153624 | 3064 | 1.53±0.39 |
|  | LA2 (n=05) | 501-1000 | 5.18 | 231758 | 4.30±0.36 | 217169 | 4094 | 1.83±0.18 |
|  | LA3 (n=01) | 1001-1500 | 0.48 | 24124 | 4.98±0.0 | 22533 | 457 | 1.89±0.0 |
|  | LA4 (n=02) | 1501-2000 | 9.12 | 401518 | 3.05±1.72 | 378373 | 12570 | 2.21±0.98 |
|  | LA5 (n=02) | 2001-2500 | 0.84 | 67249 | 7.86±1.27 | 63997 | 1301 | 1.91±0.11 |
|  | Total LA (n=15) | < 2500 | 18.09 | 887854 | 6.15±0.88 | 835696 | 21486 | 1.79±0.17 |
|  | HA (n=01) | ≥ 2500 | 0.26 | 16307 | 6.07±0.0 | 15407 | 329 | 2.01±0.00 |
| **V.** | **COLOMBIA** | | | | | | | |
|  | LA1 (n=11) | 0-500 | 12.27 | 449038 | 3.04±0.54 | 424459 | 14781 | 2.78±0.33 |
|  | LA2 (n=14) | 501-1000 | 34.57 | 879671 | 3.72±0.40 | 841542 | 24166 | 3.15±0.23 |
|  | LA3 (n=04) | 1001-1500 | 6.84 | 174719 | 3.23±0.60 | 165749 | 5190 | 2.83±0.33 |
|  | LA4 (n=0) | 1501-2000 | NA | NA | NA | NA | NA | NA |
|  | LA5 (n=0) | 2001-2500 | NA | NA | NA | NA | NA | NA |
|  | Total LA (n=29) | < 2500 | 53.69 | 1550919 | 3.40±0.29 | 1477266 | 45133 | 2.96±0.17 |
|  | HA (n=01) | ≥ 2500 | 0.86 | 21569 | 2.43±0.0 | 20239 | 808 | 3.74±0.0 |
| **VI.** | **CENTRAL ASIA** | | | | | | | |
|  | LA1 (n=0) | 0-500 | NA | NA | NA | NA | NA | NA |
|  | LA2 (n=02) | 501-1000 | 52.74 | 302091 | 0.70±0.46 | 283859 | 3459 | 1.02±0.25 |
|  | LA3 (n=0) | 1001-1500 | NA | NA | NA | NA | NA | NA |
|  | LA4 (n=01) | 1501-2000 | 6.60 | 86755 | 1.31±0.00 | 83766 | 1480 | 1.70±0.00 |
|  | LA5 (n=01) | 2001-2500 | 9.69 | 13308 | 0.13±0.00 | 13218 | 90 | 0.67±0.00 |
|  | Total LA (n=04) | < 2500 | 69.04 | 402154 | 0.71±0.30 | 380843 | 5029 | 1.10±0.23 |
|  | HA (n=0) | ≥ 2500 | NA | NA | NA | NA | NA | NA |
| **VII.** | **ITALY** |  |  |  |  |  |  |  |
|  | LA1 (n=13) | 0-500 | 38.31 | 1761536 | 4.42±1.28 | 1362913 | 47728 | 2.74±0.74 |
|  | LA2 (n=07) | 501-1000 | 21.91 | 1511860 | 7.04±1.85 | 1268831 | 55285 | 3.26±0.68 |
|  | LA3 (n=0) | 1001-1500 | NA | NA | NA | NA | NA | NA |
|  | LA4 (n=1) | 1501-2000 | 0.12 | 8414 | 6.69 | 7626 | 419 | 4.97 |
|  | LA5 (n=0) | 2001-2500 | NA | NA | NA | NA | NA | NA |
|  | Total LA (n=27) | < 2500 | 60.36 | 3281810 | 5.40±0.43 | 2639370 | 103432 | 3.02±0.19 |
|  | HA (n=0) | ≥ 2500 | NA | NA | NA | NA | NA | NA |
| **VIII.** | **SPAIN** |  |  |  |  |  |  |  |
|  | LA1 (n=11) | 0-500 | 31.96 | 1956909 | 6.08±1.81 | 80398 | 38595 | 1.80±0.46 |
|  | LA2 (n=08) | 501-1000 | 14.97 | 1249207 | 7.52±1.83 | 69978 | 34198 | 2.84±0.61 |
|  | LA3 (n=0) | 1001-1500 | NA | NA | NA | NA | NA | NA |
|  | LA4 (n=0) | 1501-2000 | NA | NA | NA | NA | NA | NA |
|  | LA5 (n=0) | 2001-2500 | NA | NA | NA | NA | NA | NA |
|  | Total LA (n=19) | < 2500 | 46.94 | 3206116 | 6.69±0.45 | 150376 | 72793 | 2.24±0.17 |
|  | HA (n=0) | ≥ 2500 | NA | NA | NA | NA | NA | NA |
| **IX.** | **BRAZIL** |  |  |  |  |  |  |  |
|  | LA1 (n=21) | 0-500 | 125.85 | 6881183 | 6.64±0.56 | 5980998 | 172846 | 2.32±0.20 |
|  | LA2 (n=06) | 501-1000 | 85.89 | 4812655 | 7.28±1.06 | 4345377 | 111929 | 1.98±0.23 |
|  | LA3 (n=0) | 1001-1500 | NA | NA | NA | NA | NA | NA |
|  | LA4 (n=0) | 1501-2000 | NA | NA | NA | NA | NA | NA |
|  | LA5 (n=0) | 2001-2500 | NA | NA | NA | NA | NA | NA |
|  | Total LA (n=27) | < 2500 | 211.75 | 11693838 | 6.78±0.48 | 10326375 | 284775 | 2.24±0.16 |
|  | HA (n=0) | ≥ 2500 | NA | NA | NA | NA | NA | NA |
| **X.** | **MEXICO** |  |  |  |  |  |  |  |
|  | LA1 (n=08) | 0-500 | 18.35 | 217178 | 1.53±0.35 | NA | 22044 | 10.75±1.41 |
|  | LA2 (n=08) | 501-1000 | 34.16 | 476865 | 1.48±0.21 | NA | 46160 | 10.25±1.16 |
|  | LA3 (n=07) | 1001-1500 | 29.31 | 347044 | 1.28±0.17 | NA | 39597 | 11.39±1.14 |
|  | LA4 (n=05) | 1501-2000 | 15.21 | 278211 | 1.79±0.24 | NA | 24094 | 9.53±1.69 |
|  | LA5 (n=03) | 2001-2500 | 19.76 | 268404 | 1.44±0.11 | NA | 34628 | 11.53±1.44 |
|  | Total LA (n=31) | < 2500 | 116.80 | 1587702 | 1.49±0.11 | NA | 166523 | 10.65±0.59 |
|  | HA (n=01) | ≥ 2500 | 9.20 | 587760 | 6.38±0.00 | NA | 29385 | 4.99±0.00 |
| **XI.** | **PERU** |  |  |  |  |  |  |  |
|  | LA1 (n=06) | 0-500 | 14.01 | 846909 | 5.27±0.65 | NA | 29303 | 3.74±0.59 |
|  | LA2 (n=05) | 501-1000 | 2.83 | 164051 | 7.50±1.96 | NA | 6287 | 3.33±0.73 |
|  | LA3 (n=01) | 1001-1500 | 0.89 | 31345 | 3.48±0.0 | NA | 923 | 2.94±0.0 |
|  | LA4 (n=04) | 1501-2000 | 2.13 | 136774 | 10.02±3.03 | NA | 5433 | 3.77±0.58 |
|  | LA5 (n=04) | 2001-2500 | 2.90 | 140571 | 5.48±1.16 | NA | 4879 | 3.20±0.39 |
|  | Total LA (n=20) | < 2500 | 22.78 | 1319650 | 6.73±0.87 | NA | 46825 | 3.50±0.27 |
|  | HA (n=05) | ≥ 2500 | 1.30 | 107414 | 11.50±2.91 | NA | 2505 | 2.39±0.09 |
| **XII.** | **BOLIVIA** |  |  |  |  |  |  |  |
|  | LA1 (n=01) | 0-500 | 0.15 | 1365 | 0.88±0.0 | 117 | 117 | 8.5±0.0 |
|  | LA2 (n=02) | 501-1000 | 3.85 | 37427 | 1.02±0.7 | 1217 | 1258 | 4.2±1.24 |
|  | LA3 (n=0) | 1001-1500 | NA | NA | NA | NA | NA | NA |
|  | LA4 (n=01) | 1501-2000 | 0.58 | 2825 | 0.48±0.0 | 76 | 80 | 2.83±0.0 |
|  | LA5 (n=0) | 2001-2500 | NA | NA | NA | NA | NA | NA |
|  | Total LA (n=04) | < 2500 | 4.58 | 41617 | 0.85±0.13 | 1410 | 1455 | 4.97±1.34 |
|  | HA (n=05) | ≥ 2500 | 9.77 | 26664 | 0.30 ±0.08 | 1125 | 1192 | 5.46±0.08 |
| **XIII** | **CHINA** |  |  |  |  |  |  |  |
|  | LA1 (n=19) | 0-500 | 921.85 | 95077 | 0.01±0.009* | 89831 | 4805 | 1.06±0.36 |
|  | LA2 (n=04) | 501-1000 | 183.54 | 2874 | 0.001±0.001** | 2851 | 18 | 0.53±0.19 |
|  | LA3 (n=03) | 1001-1500 | 99.98 | 1077 | 0.001±0.0003* | 1059 | 06 | 0.72±0.32 |
|  | LA4 (n=02) | 1501-2000 | 55.18 | 309 | 0.0007±0.0003* | 305 | 02 | 0.42±0.42 |
|  | LA5 (n=03) | 2001-2500 | 134.65 | 2104 | 0.01±0.007* | 2057 | 08 | 0.56±0.25 |
|  | Total LA (n=31) | < 2500 | 1395.2 | 101441 | 0.010±0.050* | 96103 | 4839 | 0.87±0.22 |
|  | HA (n=02) | ≥ 2500 | 9.47 | 125 | 3.41E-09±0.001 | 124 | 01 | 9.35E-07±0.46 |
| **XIV.** | **COLORADO** | |  |  |  |  |  |  |
|  | LA1 (n=0) | 0-500 | NA | NA | NA | NA | NA | NA |
|  | LA2 (n=0) | 501-1000 | NA | NA | NA | NA | NA | NA |
|  | LA3 (n=15) | 1001-1500 | 0.29 | 31090 | 11.91±2.12 | NA | 707 | 2.35±0.27 |
|  | LA4 (n=10) | 1501-2000 | 3.47 | 269840 | 8.06±1.07 | NA | 3470 | 1.17±0.12 |
|  | LA5 (n=16) | 2001-2500 | 1.76 | 115945 | 6.62±0.49 | NA | 1675 | 1.52±0.30 |
|  | Total LA (n=31) | < 2500 | 5.53 | 416875 | 8.91±0.90 | NA | 5852 | 1.74±0.17 |
|  | HA (n=23) | ≥ 2500 | 0.35 | 28410 | 6.59±0.61 | NA | 197 | 0.77±0.12 |
| **XIV.** | **GLOBAL** | |  |  |  |  |  |  |
|  | LA1 (n=135) | 0-500 | 3545.48 | 91246389 | 2.72±0.22 | 70020309 | 1967428 | 1.98±0.21 |
|  | LA2 (n=32) | 501-1000 | 2264.65 | 7479499 | 2.05±0.51 | 6735848 | 197545 | 2.51±0.28 |
|  | LA3 (n=09) | 1001-1500 | 1735.16 | 11811315 | 1.06±0.71 | 11426027 | 173474 | 1.51±0.43 |
|  | LA4 (n=05) | 1501-2000 | 24.94 | 296228 | 4.31±2.59 | 276221 | 1460 | 1.74±0.29 |
|  | LA5 (n=02) | 2001-2500 | 139.48 | 2065574 | 0.85±0.72 | 1621087 | 181899 | 4.76±4.09 |
|  | Total LA (n=183) | < 2500 | 7709.34 | 112899005 | 2.54±0.20 | 90079492 | 2525631 | 2.07±0.17 |
|  | HA (n=02) | ≥ 2500 | 30.25 | 274533 | 0.52±0.40 | 274533 | 2066 | 0.43±0.31 |

LA, Low-altitude; HA, High-altitude; NA, Not available; Data for COVID-19 incidence and fatality rates are presented as mean ± SEM. Data sources are presented in Supplementary Table 2.

**SUPPLEMENTARY TABLE 4:** Cofactors and comorbidities evaluated in altitude-specified manner.

1. **Indian Data**

|  |  | **Cofactors** | | | | | | | **Comorbidities, %** | | | | | |
| --- | --- | --- | --- | --- | --- | --- | --- | --- | --- | --- | --- | --- | --- | --- |
|  |  | **Clinical Parameters** | | | **Substance consumption rate** | | **Pollutant** |  |  |  |  |  |  |  |
| **altitude, masl** |  | **BMI** | **Blood pressure** | **Blood sugar** | **Tobacco user** | **Alcohol** | **PM 2.5** |  | **Asthma** | **Heart disease** | **Cancer** | **Diabetes** | **Hypertension** | **Stroke** |
| 0-500 | Average | 24.72 | 0.81 | 8.11 | 24.39 | 18.33 | 40.69 |  | 1.59 | 1.11 | 0.24 | 22.67 | 25.22 | 0.54 |
|  | SE | 2.10 | 0.10 | 0.53 | 2.70 | 1.59 | 4.17 |  | 0.18 | 0.16 | 0.07 | 2.14 | 1.66 | 0.02 |
|  | N | 19 | 19 | 19 | 20 | 20 | 13 |  | 19 | 19 | 17 | 16 | 18 | 16 |
| 501-1000 | Average | 20.47 | 0.91 | 7.1 | 25.01 | 22 | 47.33 |  | 1.43 | 2.03 | 0.19 | 16.45 | 23.72 | 0.42 |
|  | SE | 2.69 | 0.19 | 0.76 | 7.66 | 2.23 | 19.42 |  | 0.16 | 0.34 | 0.04 | 2.26 | 2.47 | 0.02 |
|  | N | 6 | 6 | 6 | 6 | 6 | 3 |  | 6 | 6 | 6 | 6 | 6 | 6 |
| 1001-1500 | Average | 20.36 | 0.76 | 7.28 | 45.1 | 15.56 | 63 |  | 1.51 | 1.28 | 0.08 | 24.23 | 25.52 | 0.56 |
|  | SE | 4.65 | 0.13 | 1.06 | 16.71 | 1.75 | 6.50 |  | 0.51 | 0.25 | 0.01 | 6.79 | 4.99 | 0.12 |
|  | N | 3 | 3 | 3 | 3 | 3 | 3 |  | 3 | 3 | 3 | 3 | 3 | 3 |
| 1501-2000 | Average | 13.05 | 1.4 | 5.9 | 38.85 | 30.95 | 60 |  | 0.8 | 1.05 | 0.05 | 23.7 | 19.04 | 0.54 |
|  | SE | - | - | - | - | - | - |  | - | - | - | - | - | - |
|  | N | 1 | 1 | 1 | 1 | 1 | 1 |  | 1 | 1 | 1 | 1 | 1 | 1 |
| 2001-2500 | Average | 19.37 | 1.4 | 6.82 | 45.9 | 25 |  |  | 0.82 | 1.1 | 0.1 | 25.75 | 25.90 | 0.42 |
|  | SE | 0.32 | 0.6 | 0.62 | 2.55 | 6.15 |  |  | 0.07 | 0 | 0.05 | 6.85 | 1.35 | 0.08 |
|  | N | 2 | 2 | 2 | 2 | 2 |  |  | 2 | 2 | 2 | 2 | 2 | 2 |
| 2501-4500 | Average | 26.95 | 1.45 | 6.7 | 24.56 | 20.8 | 84 |  | 0.93 | 1.48 | 0.12 | 27.23 | 33.98 | 0.45 |
|  | SE | 1.90 | 0.45 | 0.56 | 1.02 | 2.63 | 0 |  | 0.10 | 0.99 | 0.07 | 2.73 | 0.38 | 0.05 |
|  | N | 3 | 3 | 3 | 3 | 3 | 1 |  | 3 | 3 | 2 | 3 | 3 | 3 |

Blood pressure (Systolic ≥180 mm of Hg and/or Diastolic ≥110 mm of Hg); Blood Sugar ≥140 mg/dl; N, Number; SE, Standard error; BMI, body mass index; PM2.5, Particulate matter <2.5 micrometre.

**B. Global Data**

|  |  | |  | **Cofactors, %** | | | **Comorbidities, %** | |
| --- | --- | --- | --- | --- | --- | --- | --- | --- |
| **Altitude, masl** | |  | | **Alcohol Index** | **Age above 65 yrs** | **GDP per Capita, USD** | **Cardiovascular death rate** | **Diabetes** |
| **0-501** | | **Average** | | 7.09 | 9.82 | 21711.80 | 0.24 | 7.66 |
|  | | **SE** | | 0.39 | 0.61 | 1999.67 | 0.01 | 0.36 |
|  | | **N** | | 112 | 113 | 110 | 112 | 112 |
| **501-1000** | | **Average** | | 4.86 | 6.78 | 13099.67 | 0.30 | 7.15 |
|  | | **SE** | | 0.68 | 0.98 | 2708.00 | 0.02 | 0.63 |
|  | | **N** | | 31 | 30 | 30 | 31 | 30 |
| **1001-1500** | | **Average** | | 5.47 | 3.51 | 5651.66 | 0.35 | 6.04 |
|  | | **SE** | | 1.25 | 0.46 | 1943.10 | 0.04 | 1.11 |
|  | | **N** | | 8 | 8 | 8 | 8 | 8 |
| **1501-2000** | | **Average** | | 7.4 | 5.79 | 4221.60 | 0.34 | 5.61 |
|  | | **SE** | | 1.19 | 1.84 | 1555.00 | 0.05 | 0.86 |
|  | | **N** | | 5 | 4 | 4 | 4 | 4 |
| **2001-2500** | | **Average** | | 4.9 | 5.15 | 10116.69 | 0.29 | 10.08 |
|  | | **SE** | | 1.6 | 1.69 | 7219.77 | 0.13 | 2.97 |
|  | | **N** | | 2 | 2 | 2 | 2 | 2 |
| **2501-4500** | | **Average** | | 1.3 | 5.34 | 5575.70 | 0.23 | 8.50 |
|  | | **SE** | | 0.7 | 0.46 | 3132.89 | 0.02 | 1.24 |
|  | | **N** | | 2 | 2 | 2 | 2 | 2 |

yrs, years; N, number of countries; SE, standard error, Data sources are mentioned in Supplementary table 2.

**SUPPLEMENTARY TABLE 5:** India and Global incidence and fatality versus cofactors and comorbidities with increasing altitudes.

| **A) India** | | | | | |
| --- | --- | --- | --- | --- | --- |
| **Altitude, masl r, P Incidence rate vs Cofactors, %** | | | | **Incidence rate vs Comorbidities, %** | |
|  | | **BMI** | **Blood pressure** | **Diabetes** | **Hypertension** |
| 0- 500 vs 501-1000 | r | 0.47 | −0.24 | 0.71 | 0.34 |
|  | P | 0.01 | 0.25 | 2.98E-04 | 0.09 |
| 0- 500 vs 1001-1500 | r | 0.49 | −0.27 | 0.65 | 0.35 |
|  | P | 0.02 | 0.22 | 0.003 | 0.10 |
| 0- 500 vs 1501-2000 | r | 0.49 | −0.28 | 0.72 | 0.35 |
|  | P | 0.02 | 0.23 | 0.002 | 0.12 |
| 0- 500 vs 2001-2500 | r | 0.49 | −0.25 | 0.68 | 0.35 |
|  | P | 0.02 | 0.28 | 0.003 | 0.11 |
| 0- 500 vs 2501-4500 | r | 0.49 | −0.25 | 0.71 | 0.35 |
|  | P | 0.02 | 0.27 | 0.001 | 0.10 |
| **Altitude, masl r, P Fatality rate vs Cofactors, %** | | | | **Fatality rate vs Comorbidities, %** | |
|  | | **BMI** | **Blood pressure** | **Diabetes** | **Hypertension** |
| 0- 500 vs 501-1000 | r | −0.10 | −0.11 | 0.05 | −0.28 |
|  | P | 0.63 | 0.58 | 0.80 | 0.16 |
| 0- 500 vs 1001-1500 | r | −0.03 | −0.08 | 0.23 | −0.16 |
|  | P | 0.88 | 0.70 | 0.34 | 0.47 |
| 0- 500 vs 1501-2000 | r | −0.16 | −0.18 | 0.01 | −0.32 |
|  | P | 0.49 | 0.46 | 0.96 | 0.16 |
| 0- 500 vs 2001-2500 | r | −0.15 | −0.30 | 0.13 | −0.28 |
|  | P | 0.50 | 0.19 | 0.59 | 0.21 |
| 0- 500 vs 2501-4500 | r | −0.13 | −0.06 | 0.05 | −0.32 |
|  | P | 0.53 | 0.77 | 0.84 | 0.14 |
| **B) Global** | | | | | |
| **Altitude, masl** | **r, P** | **Incidence rate vs Cofactors, %** | | **Incidence rate vs Comorbidities, %** | |
|  |  | **BMI** | **Blood**  **pressure** | **Diabetes** | **Hypertension** |
| 0- 500 vs 501-1000 | r | 0.58 | 0.09 | 0.14 | 0.34 |
|  | P | 1.41E-05 | 0.31 | 0.08 | 4.89E-05 |
| 0- 500 vs 1001-1500 | r | 0.55 | 0.09 | 0.13 | 0.33 |
|  | P | 1.23E-04 | 0.32 | 0.14 | 3.31E-04 |
| 0- 500 vs 1501-2000 | r | 0.56 | 0.09 | 0.14 | 0.31 |
|  | P | 1.17E-04 | 0.33 | 0.12 | 0.001 |
| 0- 500 vs 2001-2500 | r | - | 0.09 | 0.14 | 0.31 |
|  | P | - | 0.35 | 0.13 | 0.001 |
| 0- 500 vs 2501-4500 | r | 0.57 | 0.10 | 0.13 | 0.33 |
|  | P | 1.44E-04 | 0.32 | 0.15 | 4.81E-04 |
| **Altitude, masl** | **r, P** | **Fatality rate vs Cofactors, %** | | **Fatality rate vs Comorbidities, %** | |
|  |  | **BMI** | **Blood**  **pressure** | **Diabetes** | **Hypertension** |
| 0- 500 vs 501-1000 | r | 0.021 | 0.08 | −0.05 | -0.18 |
|  | P | 0.88 | 0.36 | 0.50 | 0.03 |
| 0- 500 vs 1001-1500 | r | 0.02 | 0.09 | −0.06 | -0.18 |
|  | P | 0.85 | 0.31 | 0.50 | 0.04 |
| 0- 500 vs 1501-2000 | r | 0.03 | 0.09 | −0.07 | -0.18 |
|  | P | 0.83 | 0.33 | 0.44 | 0.05 |
| 0- 500 vs 2001-2500 | r | - | 0.08 | −0.06 | -0.18 |
|  | P | - | 0.38 | 0.50 | 0.05 |
| 0- 500 vs 2501-4500 | r | 0.03 | 0.09 | −0.07 | -0.18 |
|  | P | 0.84 | 0.33 | 0.44 | 0.06 |

Pearson-partial correlation test was applied to estimate the r, correlation coefficient, and P-value using Statistical package for social sciences (SPSS 16.0). Significance was maintained at *P*<0.05. masl, metre above sea level; BMI, body mass index; vs,versus.
