# Supplementary figures and images for: Disparities in COVID-19 incidence and fatality rates at high-altitude
Source: PeerJ. 2023 Feb 6;11:e14473. doi: 10.7717/peerj.14473 (PMC9922493; doi:10.7717/peerj.14473)

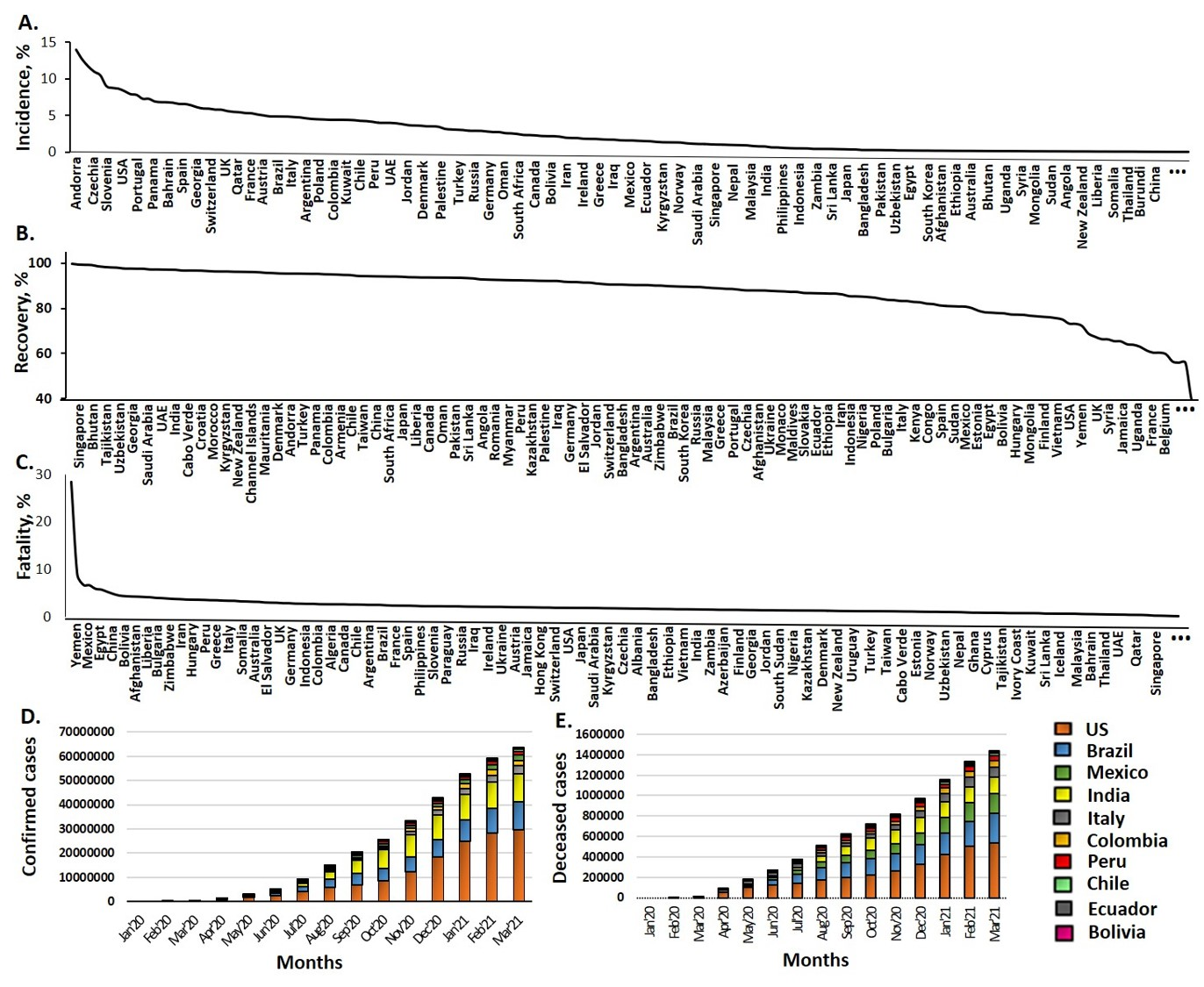

Supplement: Supplemental Information 2 — India included: (A) Incidence rate (B) Recovery rate, (C) Fatality rate, (D) Time-series of Confirmed cases, and (E) Time-series of Deceased Cases, January’2020-March-2021. …refers to few more countries that were not included due to size limitation. [file peerj-11-14473-s002.png]
